# When assessing generalisability, focusing on differences in population or setting alone is insufficient

**DOI:** 10.1186/s13063-020-4178-6

**Published:** 2020-03-20

**Authors:** Helen E. D. Burchett, Dylan Kneale, Laurence Blanchard, James Thomas

**Affiliations:** 1 Faculty of Public Health & Policy, London School of Hygiene & Tropical Medicine, London, UK; 2 EPPI-Centre, UCL Institute of Education, University College London, London, UK; 3 School of Life & Medical Sciences, University of Hertfordshire, Hatfield, UK

**Keywords:** Generalisability, Applicability, Research use, Methodology

## Abstract

Generalisability is typically only briefly mentioned in discussion sections of evaluation articles, which are unhelpful in judging whether an intervention could be implemented elsewhere, with similar effects. Several tools to assess generalisability exist, but they are difficult to operationalise and are rarely used. We believe a different approach is needed. Instead of focusing on similarities (or more likely, differences) in generic population and setting characteristics, generalisability assessments should focus on understanding an intervention’s mechanism of action - why or how an intervention was effective. We believe changes are needed to four types of research. First, outcome evaluations should draw on programme theory. Second, process evaluations should aim to understand interventions’ mechanism of action, rather than simply ‘what happened’. Third, small scoping studies should be conducted in new settings, to explore how to enact identified mechanisms. Finally, innovative synthesis methods are required, in order to identify mechanisms of action where there is a lack of existing process evaluations.

## Background

Typically, when writing up results papers from intervention evaluations, generalisability is somewhat of an afterthought; a line or two added to the end of the discussion. We often include some kind of token statement akin to, ‘this intervention could be generalisable to other low-income settings’, or ‘to similar populations’. But what is the basis of these claims? Despite the growth of the evidence-based movement, there remains surprisingly little evidence on how to assess generalisability. In contrast, more emphasis has been paid to internal validity, i.e. whether the results of a study are ‘true’, based on the study design and methods used. It is argued that initial studies should focus on small populations and have high internal validity, until causal mechanisms have been proven. Then the intervention can be scaled up to larger studies with more diverse populations and settings and greater external validity. However this distinction is less clear for complex interventions, where context and implementation are critical to the extent of an intervention’s effect [1].

In this commentary, we argue that generalisability statements in article discussion sections are unhelpful in judging whether an intervention really could be implemented in other settings or populations, with similar effects. These statements are typically based on observable similarities (or more likely, differences) in generic population and setting characteristics, regardless of whether they might be expected to influence generalisability, and are therefore restricted to describing ‘surface similarity’ [2]. We believe that a different approach is needed.

### Assessing generalisability

Establishing the parameters of where and when evidence may be generalisable is a complex undertaking. Although several frameworks and checklists have been developed to help researchers and/or decision-makers assess generalisability, none have been widely used [3, 4]. It could be argued that, unlike internal validity, generalisability is a more subjective judgement and has a tendency to be made in a less explicit manner [5]. Yet several studies demonstrate that failure to establish generalisability directly hinders evidence use in health decision-making [6, 7]. The plethora of different approaches available for assessing generalisability is not only testament to the complexity of the endeavour, but is also indicative of a lack of consensus regarding the parameters of generalisability. This applies to generalising evidence from a single study, from a systematic review or during the synthesis of studies within a systematic review.

To illustrate our argument, we’ll consider the generalisability of a weight management intervention that was found to be effective among overweight postpartum women in Gothenburg, Sweden, [8] to the English context. This intervention had three intervention arms and a control arm. The most effective arm involved a 12-week treatment programme where participants received an initial 1.5‑hour individual behaviour modification counselling session with a dietician and a 1-hour follow-up home visit in week six. In addition, participants received a dietary modification plan with advice on strategies, an electronic body scale and biweekly text messages where they were asked to report their weight.

A crude consideration of generalisability based on surface similarity may lead us to decide that while the intervention may be applicable to postpartum women, it would not be applicable to women who have not recently had a baby, or to men. If we look more closely at the study population and compare it to the English context, we might note that the former was older, more educated and more likely to breastfeed than the English postpartum population. This may lead us to conclude that it would not be applicable to this population. However, the effects of age, education and breastfeeding on the intervention may or may not be of critical importance to the intervention’s success.

If we go beyond considerations of population and look at the setting, we might conclude that the intervention is generalisable to urban settings in high-income countries, albeit ones with similar maternity leave policies and culture, comparatively low levels of income inequality, and where there is sufficient mobile phone coverage. Further questions could be asked about the feasibility of home visits, provision of free weighing scales for participants and the use of dieticians as providers. Again, we may end up judging that the contexts in Sweden and the UK are so different that the intervention is unlikely to be feasible without major adaptations, which could then alter its effectiveness.

Using existing approaches and lenses, it is easy to reach a conclusion that the intervention will not be generalisable to most other populations or settings. Indeed, as has been reported elsewhere, it is far easier to identify differences and therefore to argue that an intervention is not generalisable, than to decide that sufficient similarity exists to allow a conclusion of ‘generalisability’ [3]. A smaller risk is that we erroneously assume evidence is generalisable on the basis of similarities of characteristics that are, in fact, irrelevant to its implementation or effectiveness.

Understanding the mechanism of action - the way in which an intervention interacts with its context to lead to an effect - is critical for understandings of generalisability, but is all too frequently overlooked. Instead of searching for differences in population and setting characteristics as a starting point, generalisability assessments should focus on understanding *why* or *how* the intervention was effective. This type of mechanistic account of generalisability aims to identify patterns and processes of importance to understand how interventions lead to effects [9]. Instead of examining patterns of difference, or indeed similarity, generalisability assessments should begin with identifying mechanisms of action and modifiers of importance.

For example, in the Swedish weight loss study, semi-structured interviews were conducted with participants to explore their experiences [10]. The researchers identified a process experienced by participants who were successful in losing weight, but not by those who were unsuccessful. This process involved participants initially feeling that they were not in control of their lives and were dissatisfied with this. There was then a ‘catalytic interaction’ between the provider and participant, which depended on “individualised, concrete, specific and useful information, and an emotional bond through joint commitment, trust and accountability” (p7 [10]). Shifting from considering the characteristics of the population and setting to examining the process leading to effectiveness broadens the generalisability of the evidence beyond urban, educated, older, breastfeeding postpartum women in high-income countries. One could hypothesise that this process might also occur among men, with rural populations, or with women who were not postpartum.

### Rethinking our approach to generalisability

If we take the generalisability of processes and mechanisms as our starting point, then the types of evidence we need from effectiveness research changes. A different approach is needed if we are to improve our understanding of generalisability. Understanding how an intervention exerts its effect is critical at all stages of intervention development, evaluation and future use. Understanding an intervention’s mechanisms of action, and how these can be enacted in different contexts, should enable us to develop a clearer view of whether and how interventions could be generalizable to new contexts. Such understandings can and should be developed, evaluated and refined at all stages in the process; a priori theory development alone is unlikely to suffice. First, interventions should be developed based on a clear programme theory (e.g. theory of change) and evaluations should check that the various outcomes along their hypothesised causal pathway are being ‘triggered’ in line with their theory.

Second, we should focus on understanding how the intervention is implemented and experienced in context. We need to understand its mechanisms of action and for this we need process evaluations linked to outcome evaluations [11]. This requires a shift in the purpose of a process evaluation, so that they are not focused on reporting ‘what happened’ but also aim to develop an account of ‘how things happened’ in order to understand what the intervention’s mechanisms of action were. It also requires us to view process evaluations as a core output of a trial and not as an optional and less important component than outcome evaluations.

Third, once we’ve established how an intervention worked in its original context, e.g. what the mechanisms of action were, we can explore how to enact these mechanisms in a new context. This may be through small scoping studies, rather than a large replication trial. With the weight management example earlier, this could include identifying what is needed in order for participants to develop an emotional bond with providers.

We also need to consolidate new methods of synthesising existing literature in order to identify potential mechanisms of action, particularly in areas that lack the process evaluations proposed above. This could involve the greater use of methods such as qualitative evidence synthesis methods, [12] qualitative comparative analysis, [13] or theoretical synthesis [14] to identify potential mechanisms of action to test in future research. Logic models are increasingly used in systematic reviews [15, 16] to build mechanistic accounts of how interventions work [9] and could also be a means to assess generalisability. Logic models, which are purposively designed to elucidate the mechanisms of action and to explore how they interact with contextual factors, could represent a valuable, but hitherto underutilised, tool in exploring generalisability.

Finally, there is the issue of roles and responsibilities. If generalisability is an issue for both researchers and research users to consider, then it follows that research funding should be made available to support this work. The broader range of methods discussed above will only be used if funding is available. Funders need to recognise the value of this spectrum of methods, rather than focusing particularly on traditional outcome evaluations and systematic reviews.

## Conclusion

Overall, we believe that a better approach to the phases of research, as can be found with clinical trials, is needed in public health. An initial phase of research would involve smaller pilot studies that test out mechanisms of action, exploring *how* a given intervention may achieve its effect. Once the mechanism is identified, then larger trials, with integral process evaluations, can be conducted. Subsequently, scoping studies could be conducted to identify whether and how interventions could be generalised to new populations and/or settings.

The benefits of these modified approaches are that they explicitly encourage researchers (and research users) to theorise about the generalisability of research and develop a deeper understanding of how interventions are likely to improve health outcomes. They can identify what types of modifications may be needed for successful implementation in new settings, without reducing effectiveness. Such an approach could see the end of statements about generalisability that are reflections of surface similarity, and to actually provide a more useful understanding of an intervention [2]. Our approach would see ‘generalisability’ becoming less of an afterthought and more of an integral component of research.

## Data Availability

Not applicable.
